# Enhancing the porosity of chitosan sponges with CBD by adding antimicrobial violacein

**DOI:** 10.1016/j.heliyon.2024.e35389

**Published:** 2024-07-31

**Authors:** Dorota Chelminiak-Dudkiewicz, Magdalena Wujak, Dariusz T. Mlynarczyk, Jolanta Dlugaszewska, Kinga Mylkie, Aleksander Smolarkiewicz-Wyczachowski, Marta Ziegler-Borowska

**Affiliations:** a Department of Biomedical Chemistry and Polymer Science, Faculty of Chemistry, Nicolaus Copernicus University in Torun, Gagarina 7, 87-100, Torun, Poland; b Department of Medicinal Chemistry, Faculty of Pharmacy, Collegium Medicum in Bydgoszcz, Nicolaus Copernicus University in Torun, Jurasza 2, 85-089, Bydgoszcz, Poland; c Chair and Department of Chemical Technology of Drugs, Poznan University of Medical Sciences, Rokietnicka 3, 60-80, Poznan, Poland; d Department of Genetics and Pharmaceutical Microbiology, Poznan University of Medical Sciences, Rokietnicka 3, 60-806, Poznan, Poland

**Keywords:** Porous chitosan sponge, Violacein, Physicochemical properties, Wound dressing

## Abstract

Given the growing interest in non-toxic materials with good anti-inflammatory and antimicrobial mechanical properties, this work focuses on preparing chitosan sponges with violacein and cannabis oil crosslinked with dialdehyde chitosan. The sponge was tested for its physicochemical and biological properties, presenting a high swelling rate, good thermal stability, and satisfactory mechanical properties. The obtained sponge's water vapor transmission rate was 2101 g/m^2^/day and is within the recommended values for ideal wound dressings. Notably, adding violacein favorably affected the material's porosity, which is essential for dressing materials. In addition, studies have shown that the designed material interacts with human serum albumin and exhibits good antioxidant and anti-inflammatory properties. The antibacterial properties of the prepared biomaterial were assessed using the Microtox test against *A. fisherii* (Gram-negative bacterium) and *S. aureus* (Gram-positive bacterium). The investigated material provides potential therapeutic benefits due to the synergistic action of chitosan, violacein, and cannabis oil so that it could be used as a dressing material. The natural origin of the substances could provide an attractive and sustainable alternative to traditional commercially available dressings.

## Highlights

• A chitosan sponge (CS) with violacein (Viol) and cannabis oil (CanO), crosslinked with dialdehyde chitosan, was obtained.
• (CanO-Viol)CS sponge is highly absorbent, biocompatible with mouse fibroblasts, and has good antimicrobial properties.
• The addition of violacein increased the porosity of the sponge.
• (CanO-Viol)CS sponge is recommended for biomedical applications, especially as wound dressing material.

## Graphical abstract

**Figure:**
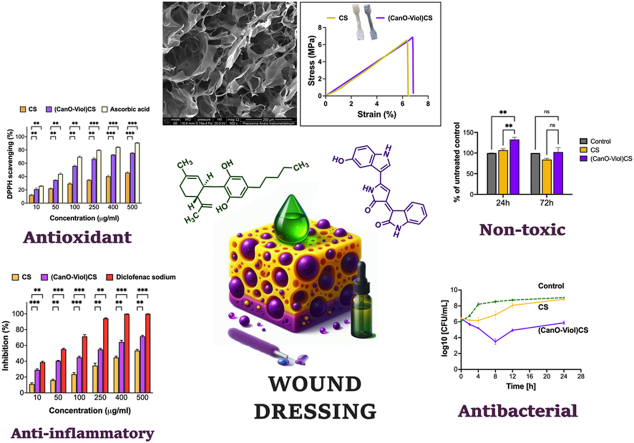

## 1 Introduction

The skin is the human body's largest organ, a barrier to the environment [1]. Skin damage interferes with proper functioning, increasing the probability of wound infection. Therefore, it is essential to quickly protect the wound with an appropriate dressing to prevent infection, speed up wound healing, and reduce the number of complications. A good wound dressing should meet many conditions, such as adequate moisture content (which will protect the wound from dehydration), biocompatibility, degradability, and non-toxicity [2,3]. Moreover, the wound dressing should have sufficient mechanical properties: it should be strong enough to withstand physical stress and, at the same time, soft enough to support the patient's regular activity. Porous sponges based on natural and synthetic polymers are widely used in biomedical dressings [4]. However, synthetic polymers could cause side effects that are highly unfavorable to patients' health and safety. Therefore, more and more attention is being paid to the use of biopolymers.

Many biomaterials can be used as a base for dressing materials, such as collagen, cellulose, dextran, starch, etc. However, chitosan (CS) is an ideal choice for wound dressings for all biopolymers due to its exceptional biocompatibility, antioxidant activity, and biodegradability [[5], [6], [7], [8], [9]]. In addition, CS has been shown to promote tissue growth and differentiation during wound healing [10]. Shen et al. [11] obtained chitosan-based hydrogel by using a multilayered upconversion nanocomposite constructed by the upconversion nanoparticles coated with a mesoporous silica-loaded zinc phthalocyanine. The results show that this wound dressing can effectively reduce inflammation and significantly accelerate wound healing due to its low toxicity, deep tissue penetration, good biocompatibility, and antibacterial solid capacity. In another study, the authors [12] designed a material using chitosan as a functional layer and a double polyacrylamide hydrogel. In turn, the protective layer of the dressing was poly (vinyl alcohol)-polyacrylamide/glycerin. The results showed that this dual dressing exhibited excellent antibacterial and cytocompatibility and could significantly accelerate skin tissue regeneration and wound closure. In another work, authors grafted CS, which was prepared and crosslinked with sodium alginate (SA) to synthesize CS-poly (MA-*co*-AA)SA hydrogel via a free radical grafting method. The prepared hydrogel demonstrated excellent effectiveness against (≥90 % inhibition) *Candida albicans* biofilms. Furthermore, hydrogel's *in vitro* wound healing outcomes indicated its potential application for chronic wound treatment [13].

Complementing the design of a good wound dressing is incorporating bioactive agents, such as antimicrobials and plant oils, into the matrix to accelerate wound healing and induce tissue formation. There are many natural oils available in the literature that are used in wound treatment. One example is thyme oil, which was introduced into a chitosan matrix by Hamedi et al. [14]. The authors showed that this material has good mechanical properties and can limit the growth of *E. coli* and *S. aureus* bacteria. Another example widely used in wound treatment is tea tree oil. Hu et al. [15] obtained a robust and antimicrobial hydrogel by Schiff base reaction between carboxymethyl chitosan and genipin, and tea tree oil was encapsulated in the hydrogel network by emulsification. The obtained hydrogels present high adhesive strength (∼162.75 kPa), excellent antibacterial properties (over 90 %), and excellent biocompatibility. Hajili et al. [16] obtained alginate nanofibers with lavender oil as a potential material for burn wound healing. Antimicrobial results showed inhibition of the nanofibers against *S. aureus*. Anti-inflammatory activity was tested using rat skin exposed to medium ultraviolet (UVB) radiation. The data suggested that the nanomaterial reduced bacterial viability.

Noteworthy is cannabis oil (CanO), which exhibits many health-promoting properties, such as antioxidant, anti-inflammatory, and anticancer properties [17,18]. The oil contains several substances (such as vitamins, minerals, and fatty acids) which can further affect wound healing. The main component of the oil is non-psychoactive cannabidiol (CBD), known for its beneficial properties [[19], [20], [21]]. In addition, recent findings suggest that cannabidiol may also be a potential drug against human coronavirus, which can be used in combination or with other drug molecules to treat patients with COVID-19 [22]. Therefore, using it in the design of a chitosan sponge may be an additional advantage. Furthermore, our previous studies suggest that chitosan material with cannabis oil shows outstanding photostability, which is crucial for biomedical applications [23].

Moreover, in this study, we decided to further enhance the antimicrobial protection of the designed dressing materials by introducing violacein (Viol) into the biopolymer matrix. Viol is a naturally occurring antimicrobial substance derived from certain strains of Gram-negative bacteria (mainly *Chromobacterium violaceum*). Notably, violacein has been shown to exhibit lower toxicity and better biocompatibility than synthetic antibiotics [[24], [25], [26]]. Besides antimicrobial properties, the essential activities of violacein include interfering with the physiological function of biological membranes, inhibiting cell proliferation, and providing antioxidant and anti-inflammatory properties [27]. In addition, Viol's antiviral activity against some developed and non-developed viruses has also been reported [28]. To our knowledge, violacein has not yet been used to design wound dressing materials.

**Fig. 1:**
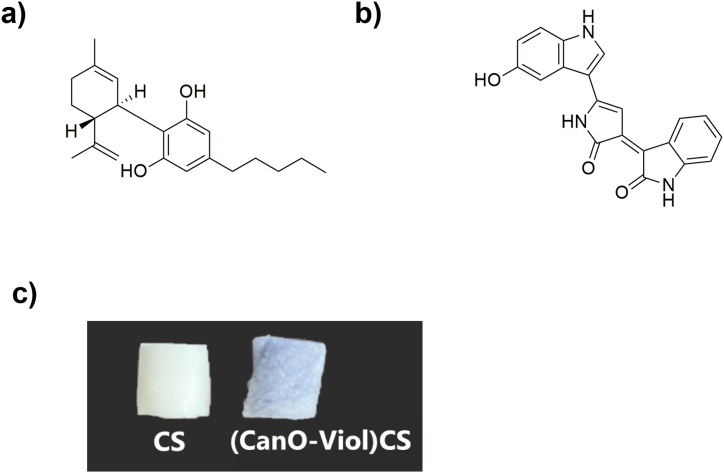
Chemical structure of a) cannabis oil and b) violacein; c) photograph of the obtained materials.

The present study obtained a highly porous chitosan sponge enriched with cannabis oil and violacein ((CanO-Viol)CS) (Fig. 1). The material was characterized in terms of its physicochemical and biological properties. Moreover, the interaction of the obtained sponge with human serum albumin (HSA) was also studied. The study showed that adding cannabis oil and violacein improved the antimicrobial properties, suggesting that the material could be potentially used as wound dressing.

## 2 Materials and methods

### 2.1 Materials

Chitosan (low molecular weight, deacetylation degree of 79 %), phosphate buffer saline (PBS) (pH 7.4), diclofenac sodium, human serum albumin (HSA), bovine serum albumin (BSA), lysozyme, and 2,2-diphenyl-1-picrylhydrazyl radical (DPPH) were purchased from Sigma-Aldrich (Munich, Germany). Violacein was purchased from Immuno Life. Acetic acid (99.8 %), sodium hydroxide, hydrochloric acid, and phenolphthalein were purchased from Avantor Performance Materials Poland (Gliwice, Poland). Cannabis oil (20 % of CBD in oil; CBD Pure) was purchased from a local pharmacy. Water was purified with a Milli-Q Water Purification System (Millipore Corp., Bedford, MA).

Reagents for the antibacterial tests: Microbank cryogenic vials (ProLabDiagnostics, Canada), Brain Heart Infusion Broth (Oxoid, UK), Tryptone Soya Agar (Oxoid, UK), Sodium chloride (Avantor Performance Materials Poland S.A., Poland).

Reagents for the Microtox test: Microtox® Acute Reagent, Microtox® Diluent, Microtox® Osmotic Adjusting Solution, Microtox® Reconstitution Solution and disposable glass cuvettes were purchased from Tigret sp. Z o. o. (Warszawa, Poland).

Reagents for the *in vitro* fibroblast test: Eagle's Minimum Essential Medium (EMEM), fetal bovine serum (FBS), Non-Essential Amino Acids (NEAA), l-glutamine, trypsin-EDTA, penicillin/streptomycin solution, and Dulbecco's phosphate buffer saline (DPBS) were purchased from Biowest (Nuaillé, France). 2-Isopropanol, hydrochloric acid, and MTT (3-(4,5-dimethylthiazol-2-yl)-2,5-diphenyltetrazolium bromide; suitable for cell culture) were purchased from Sigma-Aldrich. All reagents were used without additional purification.

### 2.2 Synthesis of the dialdehyde chitosan (DCS)

Dialdehyde chitosan was synthesized according to our previous work [29]. Briefly, chitosan (1.0 g) was dissolved in an acetic acid solution (C = 1 %, 100 mL) and then oxidized with sodium periodate (0.7 M) using a magnetic stirrer in the dark (40 °C, 3h). After cooling to room temperature, 40 mL of acetone was added. The precipitated dialdehyde chitosan was filtrated and washed three times with deionized water. The white product was dried at room temperature in the darkroom for 24 h. The reaction yield for obtaining dialdehyde chitosan was 0.91 m/m.

### 2.3 Synthesis of the chitosan sponge with cannabis oil and violacein cross-linked with dialdehyde chitosan ((CanO-Viol)CS)

First, chitosan (CS) (1.0 g) was dissolved in an acetic acid solution (C = 1 %, 100 mL) using a mechanical stirrer (1 h, room temperature, 1000 rpm). Next, 0.01 g of cannabis oil (CanO) (1 % by weight to chitosan) was added to the CS solution, and stirring was continued for 30 min. After this, violacein (Viol) (C = 1 %, 1 mL) was added at 0.2 mg per 1 mL of solution. The mixture was stirred at 25 °C for 1.5 h with a magnetic stirrer (800 rpm). Then, glycerin was added at 0.05 g per 1 mL of solution as a plasticizer and continued for 1.5 h (800 rpm, 25 °C). As a cross-linking agent, dialdehyde chitosan was added (0.05 g), and the mixture was stirred for 1.5 h at room temperature. After this, the solution was poured into a 24-well plate, frozen (−20 °C), and lyophilized (Labconco, 48 °C; 0.031 mBar, 48 h). The obtained sponges were removed from the plate and labeled as (CanO-Viol)CS.

### 2.4 Determination of the cross-linking degree

The degree of cross-linking of the (CanO-Viol)CS sponge was determined according to the previously reported method [30]. First, the sponge was weighed (m_0_). After this, the sample was placed in a flask containing acetic acid (40 mL, C = 1 %) and extracted (70 °C, 24 h). The insoluble residue was separated and dried at 60 °C in a vacuum for 24 h. After this time, the sample was re-weighed (m_1_). The analysis was repeated three times. The degree of cross-linking was calculated using the following formula:

$$Cross-linking\;Degree\;\left(\%\right)=\frac{m_{1}}{m_{0}}*100\%$$

### 2.5 Characterization of the physicochemical properties of the chitosan sponge with cannabis oil and violacein ((CanO-Viol)CS)

#### 2.5.1 Attenuated total reflectance spectroscopy (ATR-FTIR)

ATR-FTIR spectra of chitosan, cannabis oil, violacein, and the obtained (Can-Viol)CS sponge were recorded under ambient conditions using a Spectrum-Two spectrophotometer (PerkinElmer, Waltham, MA, USA) equipped with a diamond crystal. Spectra were recorded in the 4000-400 cm^−1^ range, with a resolution of 16 cm^−1^ and 64 scans. After recording the spectra, the baseline and ATR corrections were made.

#### 2.5.2 Scanning electron microscopy (SEM)

The morphology of the CS sponge and (CanO-Viol)CS sponge was examined using a scanning electron microscope (1430 V P LEO Electron Microscopy Ltd.). The samples were sputtered with gold before measurement.

#### 2.5.3 Porosity

The porosity of the CS sponge and (CanO-Viol)CS sponge was characterized using ethanol displacement [31]. The samples (0.2 g) were immersed in ethanol (4 mL) for 1 h. After that, samples were removed from the ethanol, and the excess ethanol on the surface was removed with filter paper. The porosity of the sponges was calculated using the following formula:

$$Porosity\;\left(\%\right)=\frac{\left({W-W}_{0}\right)}{\rho *V}*100\%$$

where W_0_ and W are the weight of the sponge before and after immersing in ethanol, respectively, ρ represents the density of absolute ethanol (0.7893 g/cm^3^ at 20 °C), and V denotes the volume of the sponge.

#### 2.5.4 Thermogravimetric analysis (TGA)

Thermogravimetric analysis of CS and (CanO-Viol)CS sponges was carried out on a TA Instruments thermogravimetric analyzer (SDT 2960 Simultaneous DSC-TGA) at a heating rate of 10 °C/min from ambient to 600 °C in a nitrogen atmosphere.

#### 2.5.5 Mechanical properties

The mechanical properties of CS and (CanO-Viol)CS sponges were tested using a Shimadzu EZ-Test E2-LX testing machine (Shimadzu, Kyoto, Japan). Five paddle-shaped samples were prepared to conduct tensile tests. The mechanical properties of the sponges were measured at a speed of 20 mm/min at room temperature. At least three repetitions were performed for each sample.

### 2.6 Water vapor transmission rate (WVTR)

The water vapor transmission rate (WVTR) was carried out on sponges to evaluate their gas exchange capacity [32]. A sponge with a diameter of 40 nm was prepared for this study. A solution containing chitosan (CS) or a mixture of chitosan with cannabis oil and violacein ((Can-Viol)CS) was poured into a 40 mm diameter plastic Petri dish, frozen and freeze-dried. Deionized water (5 mL) was poured into an empty plastic box (40 mm diameter). Its opening was closed with a CS sponge or a (Can-Viol)CS sponge. The box was kept at 37 °C for 24 h, and the evaporated water through the samples was measured using the following equation:

$$WVTR\;\left(\frac{\frac{g}{m^{2}}}{h}\right)=\frac{\left(\frac{\Delta w}{\Delta t}\right)}{A}$$

Where $\left(\frac{\Delta w}{\Delta t}\right)$ denoted the slope of the plot and A denoted the effective transfer area.

### 2.7 Swelling analysis

The swelling ratio of the sponges was tested using swelling tests, in which previously weighted dry sponges were immersed in PBS buffer (pH = 7.4, 10 mL), and incubated in a thermomixer at 37 °C. The samples were removed from the solution at different time intervals (1, 2, 3, 4, 5, 6, 24, and 48 h). Excess water was removed from the sponge surface using filter paper and re-weighted. At least three repeats were performed for each sample. The swelling ratio was calculated according to the formula:

$$Swelling\;rate\;\left(\%\right)=\frac{\left(W_{s}-W_{D}\right)}{W_{D}}*100\%$$

where W_S_ is the weight of the swollen sponge and W_D_ is the weight of dried sponge [33].

### 2.8 Biodegradation analysis

The biodegradability of CS and (CanO-Viol)CS sponges was determined by monitoring their weight changes over 14 days [34]. A sponge of known mass (m_0_) was incubated in a PBS solution (pH = 7.4, 37 °C, 10 mL) containing lysozyme (0.5 mg/mL) solution. After each day, the sponge was removed from the solution and re-weighed (m). The measurement was performed three times. The biodegradability of the samples was calculated using the following formula:

$$Biodegradation\;\left(\%\right)=\frac{\left(m_{0}-m\right)}{m_{0}}*100\%$$

### 2.9 Antioxidant properties

A DPPH assay was used to test the antioxidant properties of the materials. The sponge (10–500 μg/mL) was incubated with 3 mL of 1 mM ethanolic solution of 1,1-diphenyl-2-picrylhydrazyl (DPPH) at room temperature in the dark. Blank DPPH without a sample was used as a control group. The absorbance of the resulting solutions and the blank were recorded after 30 min at 517 nm at room temperature using a UV–Vis spectrophotometer (UV-1800 spectrophotometer, Shimadzu, Japan). The measurement was performed three times. The radical scavenging potential of the samples was measured using the following formula:

$$DPPH\;scavening\;\left(\%\right)=\frac{\left(A_{0}-A_{S}\right)}{A_{0}}*100\%$$

where A_0_ is the absorbance of the DPPH solution, and A_s_ is the absorbance of the sponge [35].

### 2.10 Anti-inflammatory properties

The anti-inflammatory properties of CS and (CanO-Viol)CS sponges were determined by inhibiting the denaturation of bovine serum albumin (BSA) by the materials obtained. BSA solution (5 mL, 5 %) was incubated with the sponge (10–500 μg/mL) at 37 °C for 15 min on a thermomixer (200 rpm). The sample was then heated in a water bath at 70 °C for 5 min. Diclofenac sodium salt (10–500 μg/mL) was used as a model anti-inflammatory compound. The absorbance of the obtained mixtures and the model solution was measured spectrophotometrically (UV–Vis spectrophotometer, Shimadzu, Japan) at 278 nm. The experiment was performed three times. The percentage of denaturation inhibition was calculated using the following formula:

$$Inhibition\;\left(\%\right)=\frac{\left(1-A_{s}\right)}{A_{c}}*100\%$$

where, A_c_ is the absorbance of the control and A_s_ is the absorbance of the sponge [36].

### 2.11 Protein adsorption

The evaluation of the adsorption capacity of human serum albumin (HSA) by the sponges obtained was measured using a spectrofluorometer, according to the method described previously [18]. Briefly, CS and (CanO-Viol) CS sponges were incubated in HSA solution (6.24 μM) in phosphate buffer (50 mM, pH = 7.4) at 37 °C (200 rpm) at different time intervals (1, 5, 10, 30, 60, 120, and 1440 min). After incubation, fluorescence spectra at 285 nm excitation were recorded using a Jasco FP-8300 spectrofluorometer. The parameters of the recorded fluorescence spectra were as follows: scanning speed - 100 nm/min, Em/Ex bandwidth - 2.5 nm/5 nm, and recording range - 285–400 nm.

### 2.12 Biological properties

#### 2.12.1 Antibacterial test

The Gram-positive bacterium *Staphylococcus aureus* ATCC 29213 was used to test antimicrobial properties. Bacterial strains were stored in Microbank cryogenic vials (ProLabDiagnostics, Canada) at −70 °C ± 10 °C and cultured on Tryptic Soy Agar (TSA). Test strains were cultured aerobically in Brain Heart Infusion Broth (BHI) at 36 °C ± 1 °C for 18–20 h. The microorganisms were then collected by centrifugation (3000 rpm for 15 min), resuspended in 0.85 % sterile saline, and diluted in BHI broth to a final concentration of about 10^8^ CFU/mL. To 1 mL of BHI broth, 15 mg of sponge was added. The tubes were pre-incubated for 30 min at 20 °C and inoculated with 10 μL of bacterial inoculum (10^8^ CFU/mL). The growth control group was a tube without a sponge. At predetermined time points (0, 2, 4, 8, 12, and 24 h) after incubation at 36 ± 1 °C, 100 μL sponges were collected, and the number of viable cells (CFUs/mL) in each sample was determined using a standard plate count. The time-killing curves were studied by plotting Log CFU/mL against the time. Experiments were conducted in triplicate.

#### 2.12.2 Microtox test

The acute toxicity studies were conducted using Microtox M500 apparatus and Modern Water Microtox Omni 4.2 software. Two experiments were performed based on previously published procedures [29,37]. The first set of tests included the evaluation of the effect exerted by whole fragments of the material. In this case, a modified 81.9 % Screening test procedure was applied. Instead of the tested sample, Modern Water Diluent (2 % NaCl solution) was added to the bacterial suspension, immediately immersing a film fragment. For the second set of tests, a 40 mg film fragment was mixed in 100 mL of deionized water for 20 h, after which the resulting suspension was filtered through membrane filters (0.22 μm). The filtrate was subjected to a standard 81.9 % Screening test. All the experiments were performed in triplicate.

#### 2.12.3 Cell viability test

L929 mouse fibroblasts (NCTC clone 929: ECACC no. 88102702), purchased from the European Collection of Authenticated Cell Cultures (Salisbury, UK) were cultured in EMEM medium supplemented with 2 mM l-glutamine, 1 % NEAA, 10 % FBS and antibiotics (100 U/mL penicillin and 100 μg/mL streptomycin) at 37 °C in a humidified atmosphere with 5 % CO_2_. The cell culture was supplemented with fresh medium twice or thrice weekly. We used cells in passages 6 to 8 in the experiments. Cell viability was evaluated according to ISO 10993–5 using two methods (direct contact test and extract test). CS and (CanO-Viol)CS sponges were sterilized with UV light for 40 min in a laminar chamber and used for experiments on the same day. For 24-h and 72-h tests with extracts, L-929 cells were seeded into 96-well plates at a density of 1 × 10^4^ and 2.5 × 10^3^ cells per well, respectively, and allowed to grow for 24 h.

Extracts were prepared by incubating the sponge with 4 mL of growth medium for 24 h at 37 °C in a humidified atmosphere with 5 % CO_2_. The medium was then replaced with 100 μL of medium containing extract or medium without extract (untreated control). By the recommendations of ISO 10993–5, no additional processing (e.g., filtration, centrifugation) of the obtained extracts was performed before their application to fibroblast cells. In the direct assay, 3 × 10^5^ cells were seeded into a well of 96-well plates and allowed to grow for 24 h. The medium was then changed to a fresh cell culture medium, and one sponge was placed on the well and incubated with the cells for 24 h.

MTT assay was used to assess the cell viability. After 24 or 72 h of treatment, the medium was removed, and 100 μL of 0.5 mg/mL MTT prepared in fresh growth medium was added to each well of a 96-well plate. After 3 h of incubation at 37 °C, the solution was removed, and 100 μL of 0.04 M HCl-isopropanol was added. In the direct assay, aliquots of 100 μL were transferred to 96-well plates.

Absorbance was measured at 570 nm with background subtraction at 690 nm using a Synergy HT Multi-Mode Microplate Reader (BioTek Instruments, Winooski, VT, USA). The number of viable cells was calculated relative to control cells grown in a growth medium without a sponge (for the direct assay) or its extract (for the extract assay).

### 2.13 Statistical analysis

Data are expressed as mean ± standard error of the mean. Results were obtained from at least three replicates. One-way analysis of variance (ANOVA) was used to determine statistical significance, followed by Dunnett's or Tukey's post hoc test for multiple comparisons. Statistical analysis was performed using GraphPad Prism 9.2 software (GraphPad Software, San Diego, CA, USA). A *p*-value <0.05 was considered statistically significant.

## 3 Results and discussion

### 3.1 Characterization of the physicochemical properties of the chitosan sponge with cannabis oil and violacein ((CanO-Viol)CS)

**Fig. 2:**
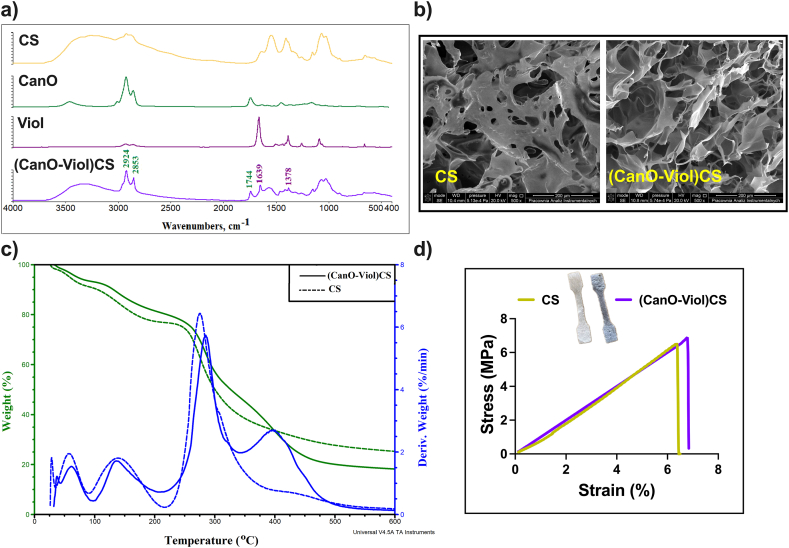
Physicochemical properties of the obtained sponges. a) ATR-FTIR spectrum, b) SEM images, c) thermogravimetric analysis, and d) mechanical properties.

Porous structures are a promising approach for applications as dressing materials. The present study obtained high-porosity chitosan materials enriched with cannabis oil (CanO) and violacein (Viol). A significant aspect of this process was the use of dialdehyde chitosan (DCS) as an effective cross-linking agent, which enabled the formation of a durable structure capable of supporting the unique properties of cannabis oil and violacein effectively. DCS has the added benefit of its natural origin. It may not have toxic effects compared to commonly used cross-linking agents (such as glutaraldehyde), which are essential for biomedical applications. The physical and chemical properties of the (CanO-Viol)CS obtained were characterized. The degree of cross-linking of the (CanO-Viol)CS sponge was 64 %. The ATR-FTIR spectrum is shown in Fig. 2a. Absorption bands attributed to OH stretching (2900-3500 cm-^1^), CH stretching (2860-3100 cm^−1^), NH bending (1560 cm^−1^), OH and CH deformation vibrations (1408, 1385 cm^−1^), and C–O–C stretching vibrations (1000-1260 cm^−1^) are present in the spectrum of pure chitosan (CS). These data are consistent with the literature [38,39]. The spectrum of cannabis oil shows characteristic bands at 3458 cm^−1^ (stretching vibrations of OH groups of cannabinoids), at 3006 cm^−1^ (CH stretching of benzene rings), and bands at 2923 cm^−1^ and 2856 cm^−1^ (corresponding to stretching vibrations of CH_3_ and CH_2_ groups of cannabinoids and fatty acid hydrocarbons). The band at 1741 cm^−1^ is associated with the presence of vibrations of the benzene skeleton [40]. The spectrum recorded for violacein shows characteristic bands at 2853 cm^−1^ (C = O amide), 1660 cm^−1^ (C = C), 1504 cm^−1^ (C–O phenol), and 1446 cm^−1^ and 1383 cm^−1^ (C–N) [41,42]. In the obtained (Can-Viol)CS sponge, bands derived from both cannabis oil (2924 cm^−1^, 2853 cm^−1^, and 1744 cm^−1^) and violacein (1639 cm^−1^ and 1378 cm^−1^) are visible, thus confirming that these substances have been successfully incorporated into the chitosan structure.

Fig. 2b shows SEM images of the CS sponge and (CanO-Viol)CS sponge. Both materials have irregular shapes and visible pores, with CS having a jagged structure. Other chitosan wound dressings, such as those formed by dissolving chitosan in an alkaline aqueous solution, followed by thermal gelling and solvent change, also exhibit a similar porous structure [43]. In addition, it can be seen that the addition of cannabis oil and violacein increased the number of pores in the material, which was confirmed by porosity analysis (33.75 % ± 3.63 and 74.54 % ± 5.96 for CS and (CanO-Viol)CS, respectively). In this case, the pores are more significant, and there are more of them compared to the base material. The increase in porosity may be due to the effective dispersion of the ingredients contained in the CanO and Viol in the material's structure, which promotes an even distribution of the pores. Our previous work obtained a levan-based sponge with cannabis oil, whose porosity was about 68 % [18]. Adding violacein had a beneficial effect on porosity, which is essential for wound healing. High porosity is desirable in dressing materials because it allows free oxygen diffusion, contributing to better tissue oxidation [44].

Thermogravimetric analysis is essential for assessing the durability and performance of potential wound dressing materials, as it allows monitoring of material degradation processes under different temperature conditions. TGA analysis was carried out for the sponge without the active substances and with the addition of cannabis oil and violacein (CanO-Viol)CS. The thermogram is shown in Fig. 2c, while the thermal parameters are summarized in Table S1 in the Supplementary Material. The thermal decomposition of chitosan occurs in three stages, consistent with the literature [[45], [46], [47]]. The first stage is associated with the release of adsorbed water. The weight loss is 14 % and 52 % for the second and third stages, respectively, related to the disruption of polysaccharide chains and the elimination of small-molecule degradation products [37]. In their study, Raj et al. also observed chitosan decomposition up to 500 °C [13]. In contrast, the thermal degradation of (CanO-Viol)CS sponge is a four-step process. The first stage, occurring with a mass loss of 7 %, is associated with water loss. The following two stages occur similarly to chitosan, except a relatively minor mass loss (34 %) was observed in the third stage. The fourth stage, appearing with a maximum temperature of 398 °C and a mass loss of 28 %, is probably related to the decomposition of cannabis oil. Nevertheless, it is worth noting that the addition of active substances affected the thermal properties of (CanO-Viol)CS. At the end of the analysis, (CanO-Viol)CS has a residue of 18 % by weight and chitosan of 28 %. It follows that the chitosan sponge enriched with cannabis oil and violacein shows more excellent thermal stability than pure chitosan, suggesting good thermal behavior, which is essential for biomedical applications.

A wound dressing should provide protection and stability around an area of injury or wound. Mechanical properties such as strength and elasticity are essential in this context. A material with sufficient strength can effectively protect the injured area from external trauma while maintaining the structural integrity of the dressing [48,49]. A tensile test was performed to determine the mechanical properties of the designed material, the engineering stress versus strain was recorded, and Young's modulus was determined. The mechanical properties of the chitosan sponge without the active substance were also studied. The results are shown in Fig. 2d and Table 1. As can be seen, adding cannabis oil and violacein affected only Young's modulus, which increased. In a study reported by Rafieian et al. it was also noted that the addition of active substances (in this case Aloe vera) led to an increase in the modulus (by almost 116 %) [50]. It may be because the chitosan fiber can be stiffened more effectively or by the presence of these components. It is known that Young's modulus of healthy skin is in the range of 4.6–20 MPa, while the tensile strength is 2.5–35 MPa [51]. Designed dressing materials should exhibit a higher value of these parameters to avoid their potential damage, even with slight wound movement. The results obtained for (CanO-Viol)CS meet this condition, suggesting that this sponge could be used as a dressing material.

**Table 1:** Mechanical properties and Water Vapor Transmission Rate (WVTR) of the obtained materials.^a^ Indicate p < 0.05 when compared to the corresponding CS.

Sample	Mechanical properties			WVTR (g/m^2^/day)
	Stress [MPa]	Strain [%]	Young's modulus [MPa]	
CS	6.31 ± 0.11	6.38 ± 0.21	133.09 ± 3.78	1983 ± 8.76
(Can-Viol)CS	6.68 ± 0.09^a^	6.79 ± 0.11^a^	154.02 ± 9.06^a^	2101 ± 13.26^a^

### 3.2 Water vapor transmission rate (WVTR)

The water vapor transmission rate (WVTR) allows the control of the permeability of the dressing to water vapor. An adequate water vapor transmission rate avoids excessive moisture retention around the wound. Excessive wetting can impede the healing process, promoting the growth of bacteria and infection [49,52,53]. According to literature data, an ideal dressing material should exhibit a WVTR value in the 2000–2500 g/m^2^/day range. The results of our study showed that the addition of active substances improved the water vapor transmission rate (Table 1). Cannabis oil and violacein may form additional spaces or pores between the fibers, allowing water vapor to flow more easily. It was confirmed by the porosity study of the materials, which showed that (CanO-Viol)CS has a much higher porosity than CS sponge (33.75 % ± 3.63 and 74.54 % ± 5.96 for CS and (CanO-Viol)CS, respectively). In addition, literature reports indicate that adding a plasticizer (in this case, cannabis oil) can increase the diffusivity of moisture through the polymer structure by increasing the distance between polymer chains [54,55]. The WVTR of the obtained sponge was 2101 g/m^2^/day and is within the recommended WVTR values for ideal wound dressings. Moreover, the WVTR of the blank sample was 2798.23 ± 6.12 g/m^2^/day. Therefore, as a wound dressing material, (CanO-Viol)CS can reduce water loss by evaporation by 25 % by providing a moist environment. Similar results were obtained in other works [56,57].

### 3.3 Swelling analysis

**Fig. 3:**
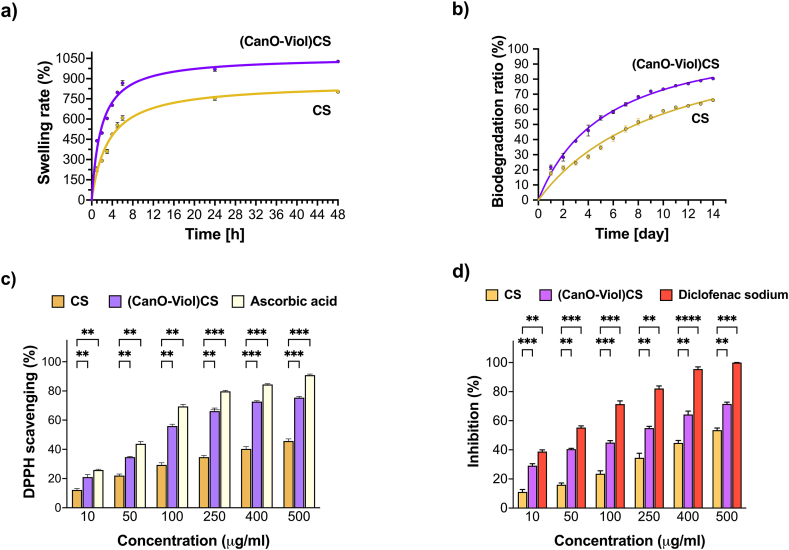
Material characterization. a) swelling rate, b) biodegradation profile, c) anti-inflammatory properties, and d) antioxidant properties of the obtained sponges.

The swelling rate is a crucial parameter, as it affects the ability of a wound dressing to maintain moisture around the wound, absorb secretions, and adjust to changing clinical conditions [[58], [59], [60]]. Therefore, when considering the design of wound dressing materials, it is essential to study the swelling rate. We conducted the study at room temperature and pH = 7.4, which allowed us to evaluate the material's response under near-physiological conditions. The results are shown in Fig. 3a. As can be seen, both the chitosan sponge and the chitosan sponge enriched with cannabis oil and violacein show a high swelling rate. However, relatively higher values were obtained for (CanO-Viol)CS (1027 % after 48 h of incubation). It is probably related to the interaction between chitosan, cannabis oil, and violacein, which may affect the structure of the sponge and its ability to retain water. Other authors have confirmed that the swelling ratio increases after adding active substances to the polysaccharide base [58,61,62]. The high values of the swelling rate of the CanO-Viol)CS sponge means that the material could effectively absorb excretions and maintain moisture around the wound.

### 3.4 Biodegradation analysis

Biodegradability plays an essential role in the design of wound dressings. A biodegradable dressing can naturally break down in the body, which reduces the need for frequent dressing changes and can help reduce the pain, stress, and risk of infection associated with regular wound dressing changes [[63], [64], [65]]. Weight loss was found to be a measure of biodegradation when the sponges were placed in PBS containing the enzyme lysozyme. Results are shown in Fig. 3b. Chitosan is known for its degradation properties, which was also confirmed in this study. After 14 days of incubation, chitosan biodegraded by 66 % under the conditions used. Most of the degradation in this biopolymer occurs in aminoglucose units, leading to further degradation of oligosaccharide units [66]. However, the addition of cannabis oil and violacein increased the weight loss by 80 %. It may be related to the porosity of the material. As mentioned earlier, the (CanO-Viol)CS sponge exhibited high porosity, which may affect enzyme penetration and facilitate biodegradation. At the same time, such a high mass loss value may be advantageous for using the obtained sponge as a potential wound dressing material.

### 3.5 Antioxidant properties

The antioxidant properties of the wound dressings may help neutralize excess reactive oxygen species (ROS), protecting cells from further oxidative stress and thereby delaying the wound healing process [67,68]. This study used 1,1-diphenyl-2-picrylhydrazyl (DPPH) free radicals to evaluate ROS scavenging activity, and ascorbic acid was used as a standard compound. As can be seen in Fig. 3c, both the CS sponge and the (CanO-Viol)CS sponge exhibit antioxidant properties. However, the sponge with active substances is a much potent radical scavenger. Cannabis oil is a rich source of natural antioxidants such as vitamin E, phytosterols, and carotenoids [69]. Hence, its presence in the (CanO-Viol)CS sponge can significantly increase the antioxidant content compared to the chitosan sponge. On the other hand, violacein is known for its antioxidant properties [70,71], which may further increase the overall antioxidant activity of the obtained material. Bölgen et al. [72] reported that chitosan cryogel incorporated with *Hypericum perforatum* oil had shown antioxidant activity. Tamer et al. [73] indicated that chitosan functionalized with polyaromatic hydroxyl molecules had a radical scavenging effect. These results also showed good similarity with our findings. The excellent antioxidant properties of the (CanO-Viol)CS sponge may make it a suitable wound dressing material.

### 3.6 Anti-inflammatory properties

Excessive inflammation can lead to further tissue damage, so controlling the inflammatory response is essential for successful healing. Wound dressings with anti-inflammatory properties can help reduce pain symptoms, which improves patient quality of life [74,75]. In our study, we used the method of denaturation inhibition of BSA protein to determine anti-inflammatory properties. We chose diclofenac sodium as a model compound, which belongs to the non-steroidal anti-inflammatory drugs. The results are shown in Fig. 3d. It can be seen that as the concentration of chitosan sponge with cannabis oil and violacein increases, increasing efficacy in inhibiting the denaturation of BSA protein is observed. Comparing the results with those obtained for diclofenac sodium, it can be seen that the (CanO-Viol)CS sponge is effective, especially at higher concentrations. However, the results are slightly lower than for the diclofenac sodium. On the other hand, CS sponge shows weaker anti-inflammatory properties than (CanO-Viol)CS. It suggested that adding cannabis oil and violacein (both of which exhibit anti-inflammatory properties) to the chitosan matrix results in the material's effectiveness in inhibiting inflammatory processes.

### 3.7 Protein adsorption

**Fig. 4:**
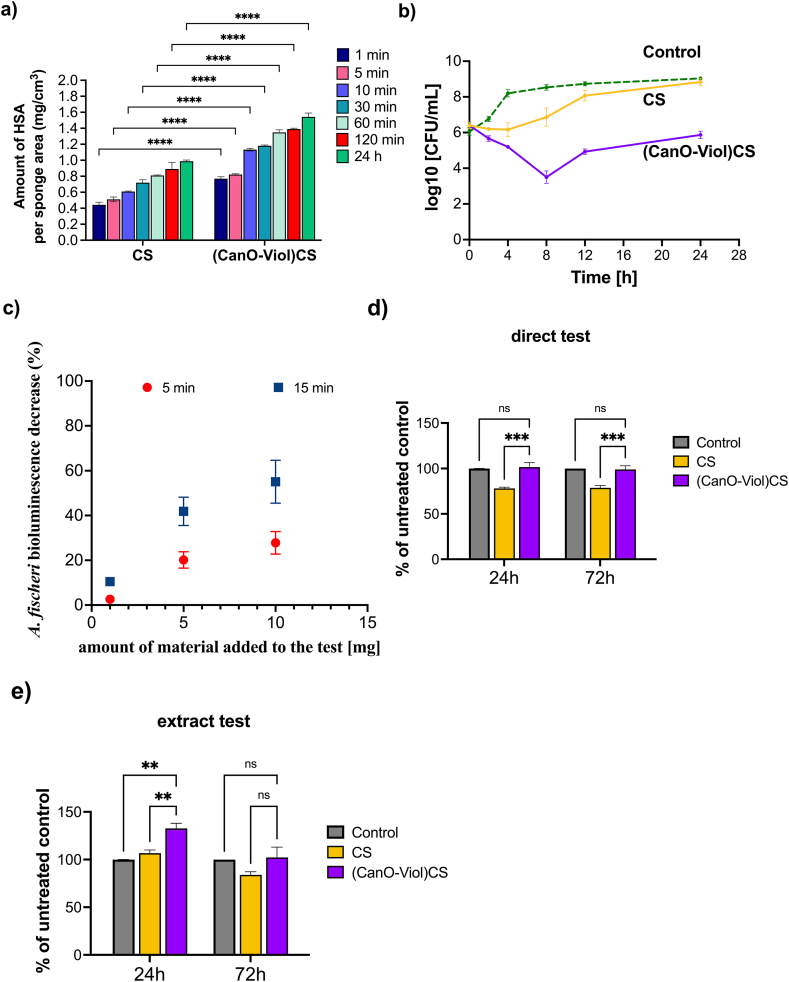
Biological properties of the obtained sponges**.** a) Protein adsorption, b) time-killing curves of *S. aureus* in the presence of the sponges, c) changes observed in the *Aliivibrio fischeri* bioluminescence decrease upon contact with the (CanO-Viol)CS sponge, and d), e) cell viability of L929 after 24 h and 72h incubation with d) sponges, and e) extracts obtained from tested sponges.

Protein adsorption regulates cell adhesion on the surface of wound dressing materials. It is essential in cell spreading, growth, and proliferation [76,77]. Protein adsorbs to the matrix surface by interacting with the functional groups present on the matrix through electrostatic interaction or van Der Waals forces [78]. This study investigated the adsorption efficiency of human serum albumin by a CS sponge and a (CanO-Viol)CS sponge. The results are shown in Fig. 4a. When comparing these two materials, it is clear that more protein is bound on the (CanO-Viol)CS sample. After 24 h of incubation, as much as 1.54 ± 0.054 g/cm^3^ of protein is attached to the obtained material, while 0.99 ± 0.015 g/cm^3^ of albumin is bound with pure chitosan. It can also be seen that the adsorption efficiency was initially faster - after 30 min, almost twice as much albumin-bound as after 1 min. The higher adsorption efficiency may be related to the better porosity of the chitosan sponge enriched with active substances. The higher protein adsorption on the obtained material may provide more cell attachment sites and may help improve cell adhesion [79].

### 3.8 Biological properties

#### 3.8.1 Antibacterial test

Gram-positive cocci, *Staphylococcus aureus* ATCC 29213, were used to evaluate the antimicrobial properties of CS and (CanO-Viol)CS sponges. These bacteria are the most common cause of acute and chronic wound infections [80,81]. The time-kill curve method was used; the results are shown in Fig. 4b. The antibacterial effect of pure chitosan against *S. aureus* was recorded only during the first 4 h of incubation. After 24h, the results were similar to the control. Adding violacein and cannabis oil to the chitosan matrix significantly increased the antimicrobial activity. According to the Clinical and Laboratory Standards Institute (CLSI), significant bactericidal activity is defined as a 3-log reduction in bacterial cells compared to the initial inoculum [82]. This criterion was met after 8h of incubation of bacteria with (CanO-Viol)CS) sponge. It was also observed that although there was a slight decrease in antimicrobial activity from 12 h of incubation of the bacteria with the sponge, a reduction in the number of bacteria compared to the initial bacterial inolocum (from 6.4 to 5.8 log10 CFU/mL) was still observed after 24 h. The alizarin nanocarriers obtained by Raji et al. in liposomes coated with chitosan and gum arabic CGL-Alz NCs also showed antimicrobial activity against *Staphylococcus aureus* [83]. The results indicate the significant antimicrobial activity of the (CanO-Viol)CS sponge, which may be a key feature in its potential use as a wound dressing material.

The (CanO-Viol)CS sponge can improve antibacterial activity through a synergistic combination of components found in the sponge. Chitosan, the sponge matrix, has natural antimicrobial properties, which in our study showed activity mainly in the first hours of incubation. Adding violacein to the chitosan matrix significantly increased the antibacterial effectiveness of the (CanO-Viol)CS sponge. Violacein is known to exhibit antimicrobial properties, and its action interferes with bacterial cellular functions, including inhibition of protein and nucleic acid synthesis. Moreover, cannabinoids present in cannabis oil, rich in anti-inflammatory and antimicrobial compounds, can act synergistically with chitosan and violacein to improve the antibacterial properties of the final material. Each (CanO-Viol)CS sponge component can act on bacteria through different mechanisms, reducing the risk of bacterial resistance and protecting the sponge from infection. In addition, studies have shown that the resulting material can help maintain a moist wound environment, which promotes tissue regeneration and inhibits bacterial growth.

#### 3.8.2 Microtox test

The prepared material was subjected to the Microtox test. The Microtox test measures the change in the bioluminescence of *Aliivibrio fischeri* (formerly *Vibrio fischeri*) bacteria upon exposure to the tested sample. The decrease is proportional to the toxic effect exerted by the tested substance or solution and, due to the test design, can be measured usually in 5–30 min upon contact of the bacteria with the tested sample [84]. A decrease of 20 % or more compared to untreated controls is considered a toxic concentration [85].

Two types of experiments were performed. Firstly, the material was subjected as it is to assess the effect it induces upon contact. The material caused a decrease in the bioluminescence in a dose-dependent manner (Fig. 4c). In all the tested concentrations, the effect was more robust after more prolonged incubation with the bacteria (15 min) than after the short incubation (5 min) with the calculated EC_50_ equal 16 mg/mL (5 min) and 8 mg/mL (15 min). The toxic effect on bacteria can probably be assigned to the antimicrobial properties of all the components of the material – all of which, chitosan [86], cannabis oil components [87,88], and violacein [89], were proven to be potent antibacterial agents and might show synergistic action [90].

Secondly, the aqueous dispersion of the material was subjected to the same test. It was to assess the potential effect associated with the material if it was to be used in wound dressings for wounds with an exudate. A 40 mg fragment of the sponge was dispersed in 100 mL of water and filtered through a 0.22 μm filter to achieve this. The resulting filtrate subjected to the test showed only negligible effect as bioluminescence decrease was equal to 3.65 % and 4.81 % after 5 and 15 min of exposure, respectively. One may assume that the activity towards *Aliivibrio fischeri* bacteria originates from the components released from the material into water, as there is almost no change in effect for the dispersed material. On the contrary, the solid material inserted directly into the bacterial suspension increased its toxic impact over time in each case, which may be attributed to the release of antibacterial molecules from the material. It may also be supported by the fact that after 5 min of incubation, the material suspension exerts almost the same effect as the 1 mg solid, while the concentration of the latter can be calculated as nearly three times as high (∼0.3 mg/mL in the dispersion vs. ∼0.9 mg/mL for the solid sample).

Although most of the reports regarding the antimicrobial effects of the tested antibiotics show a significant decrease in the bioluminescence of *A. fischeri* only for certain groups of drugs, which is supposed to be associated with the short test duration, making it impossible to act through the antibacterial molecular mechanism [91], other authors associated the bioluminescence decrease with the antimicrobial effect of complex mixtures and natural products [92]. Due to such discrepancies, the results should be taken cautiously and treated only as a possible screening of toxic effects exerted by the tested samples. However, tests performed on using *S. aureus* bacteria confirm the antimicrobial effect of the obtained sponge.

#### 3.8.3 Cell viability test

The viability of the cells on the fabricated sponges was examined using the MTT assay. A mouse fibroblast cell line (L929), often used to study wound healing *in vitro* and as an *in vitro* model to study the interaction of skin cells with dressing materials [32], was chosen. According to the recommendations of ISO standard 10.993–5, a direct contact test and an extract test were performed to test the cytotoxicity of the materials. The tests were conducted after 24 and 72 h. The results obtained in the direct contact test of cells with the tested material (Fig. 4d) showed that the CS sponge exhibited a mild toxic effect against L929 cells (cell viability 78 % of untreated control). This effect does not change after 72 h. In contrast, the (CanO-Viol)CS sponge increased cell viability compared to pure chitosan (101 % after 24h incubation). After 72h, there was a slight but statistically insignificant decrease in viability. Differences in the results obtained for the CS sponge and the (CanO-Viol)CS sponge may be related to the material's porosity. Higher porosity promotes cell adhesion and proliferation.

On the other hand, the viability of L929 cells incubated with extracts from the obtained sponges was markedly higher than that of direct contact (Fig. 4e). In this case, the viability of cells incubated for 24 h with a CS sponge increased compared to the control (106 %). After 72h, a decrease to 86 % was observed, with this value considered non-toxic. Interesting results were obtained for the chitosan sponge enriched with cannabis oil and violacein. Adding these substances increased cell viability (132 % and 102 % after 24 and 72 h, respectively). The differences in results between the direct and the extract tests may be due to the active substances' release rate. With the extract test, ingredients from the sponge can be released gradually, allowing for a more controlled effect on cells. In direct contact, cells may be exposed to higher concentrations of substances, which can lead to differences in cellular response.

However, the results of this study indicate the material's non-toxicity, suggesting that the (Can-Viol)CS sponge could be used as a wound dressing material.

**Fig. 5:**
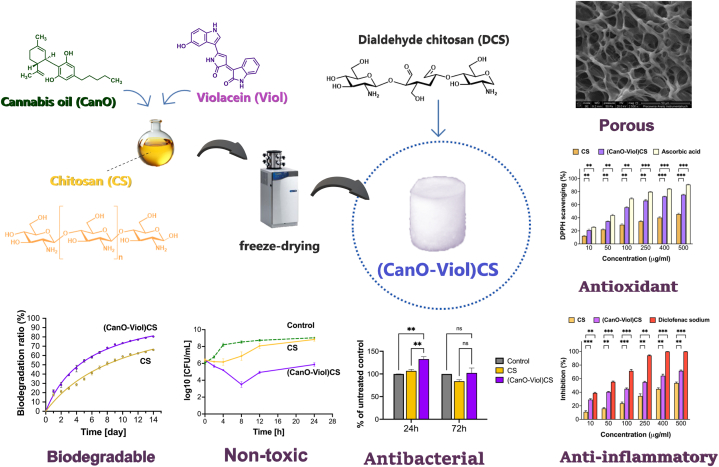
Graphic display of the main findings.

Fig. 5 contains a graphical display of the main findings of this work.

## 4 Conclusions

The designed material presented in this paper, a highly porous chitosan sponge enriched with violacein and cannabis oil (CanO-Viol)CS, was carefully evaluated from a physicochemical and biological point of view. The obtained biomaterials exhibited both anti-inflammatory and antimicrobial properties. Additionally, the (CanO-Viol)CS demonstrated an ability to interact with protein (HSA) involved in wound healing. The water vapor transmission rate of the prepared sponge was 2101 g/m^2^/day, aligning with the optimal range for wound dressings. Significantly, the inclusion of violacein enhanced the porosity of the material, a crucial factor for dressing efficacy. Furthermore, *in vitro* biocompatibility assessments indicated that the sponges were non-toxic to L929 cells. They also showed antibacterial activity against *S. aureus* and *A. fisherii* bacteria. These preliminary studies suggest that the designed biomaterial could form the basis for further *in vivo* studies for its potential use as a wound dressing material.

## Data availability statement

Data will be made available on request.

## Funding

This work was supported by the National Science Centre Poland grant UMO-2022/47/D/NZ7/01821.

## CRediT authorship contribution statement

**Dorota Chelminiak-Dudkiewicz:** Writing – original draft, Validation, Resources, Project administration, Methodology, Investigation, Funding acquisition, Formal analysis, Data curation, Conceptualization. **Magdalena Wujak:** Methodology, Investigation. **Dariusz T. Mlynarczyk:** Methodology, Investigation, Formal analysis. **Jolanta Dlugaszewska:** Methodology, Investigation. **Kinga Mylkie:** Methodology, Investigation. **Aleksander Smolarkiewicz-Wyczachowski:** Methodology, Investigation. **Marta Ziegler-Borowska:** Writing – review & editing, Supervision.

## Declaration of competing interest

The authors declare that they have no known competing financial interests or personal relationships that could have appeared to influence the work reported in this paper.

## References

[1] Hu B., Kang X., Xu S., Zhu J., Yang L., Jiang C. (2023). Multiplex chroma response wearable hydrogel patch: visual monitoring of urea in body fluids for health prognosis. Anal. Chem..

[2] Feng Y., Li X., Zhang Q., Yan S., Guo Y., Li M., You R. (2019). Mechanically robust and flexible silk protein/polysaccharide composite sponges for wound dressing. Carbohydr. Polym..

[3] Klinger C., Żółtowska-Aksamitowska S., Wysokowski M., Tsurkan M.V., Galli R., Petrenko I., Machałowski T., Ereskovsky A., Martinović R., Muzychka L., Smolii O.B., Bechmann N., Ivanenko V., Schupp P.J., Jesionowski T., Giovine M., Joseph Y., Bornstein S.R., Voronkina A., Ehrlich H. (2019). Express method for isolation of ready-to-use 3D chitin scaffolds from aplysina archeri (aplysineidae: verongiida) demosponge. Mar. Drugs.

[4] Deng C.-M., He L.-Z., Zhao M., Yang D., Liu Y. (2007). Biological properties of the chitosan-gelatin sponge wound dressing. Carbohydr. Polym..

[5] Ji M., Li J., Wang Y., Li F., Man J., Li J., Zhang C., Peng S., Wang S. (2022). Advances in chitosan-based wound dressings: modifications, fabrications, applications and prospects. Carbohydr. Polym..

[6] Hao R., Peng X., Zhang Y., Chen J., Wang T., Wang W., Zhao Y., Fan X., Chen C., Xu H. (2020). Rapid hemostasis resulting from the synergism of self-assembling short peptide and O-carboxymethyl chitosan. ACS Appl. Mater. Interfaces.

[7] Sun B., Xi Z., Wu F., Song S., Huang X., Chu X., Wang Z., Wang Y., Zhang Q., Meng N., Zhou N., Shen J. (2019). Quaternized chitosan-coated montmorillonite interior antimicrobial metal–antibiotic in situ coordination complexation for mixed infections of wounds. Langmuir.

[8] Ji M., Li J., Li F., Wang X., Man J., Li J., Zhang C., Peng S. (2022). A biodegradable chitosan-based composite film reinforced by ramie fibre and lignin for food packaging. Carbohydr. Polym..

[9] Sahdev A.K., Raorane C.J., Shastri D., Raj V., Singh A., Kim S.C. (2022). Update on modified chitosan frameworks and their applications for food, wastewater, toxic heavy metals, dyes treatment and cancer drug delivery. J. Environ. Chem. Eng..

[10] Mirhaj M., Tavakoli M., Varshosaz J., Labbaf S., Salehi S., Talebi A., Kazemi N., Haghighi V., Alizadeh M. (2022). Preparation of a biomimetic bi-layer chitosan wound dressing composed of A-PRF/sponge layer and L-arginine/nanofiber. Carbohydr. Polym..

[11] Shen J., Li L., Kang X., Lin D., Xiao Y., Yang L., Jiang C. (2023). Multilayered upconversion nanocomposite-based photodynamic hydrogel dressings for wound sterilizing and healing. ACS Appl. Nano Mater..

[12] Lu J., Fan X., Hu J., Li J., Rong J., Wang W., Chen Y., Liu W., Chen J., Chen Y. (2023). Construction and function of robust and moist bilayer chitosan-based hydrogel wound dressing. Mater. Des..

[13] Raj V., Raorane C.J., Shastri D., Kim S.C., Lee S. (2024). Engineering a self-healing grafted chitosan–sodium alginate based hydrogel with potential keratinocyte cell migration property and inhibitory effect against fluconazole resistance Candida albicans biofilm. Int. J. Biol. Macromol..

[14] Hamedi H., Moradi S., Tonelli A.E., Hudson S.M. (2019). Preparation and characterization of chitosan–alginate polyelectrolyte complexes loaded with antibacterial thyme oil nanoemulsions. Appl. Sci..

[15] Hu K., Jia E., Zhang Q., Zheng W., Sun R., Qian M., Tan Y., Hu W. (2023). Injectable carboxymethyl chitosan-genipin hydrogels encapsulating tea tree oil for wound healing. Carbohydr. Polym..

[16] Hajiali H., Summa M., Russo D., Armirotti A., Brunetti V., Bertorelli R., Athanassiou A., Mele E. (2016). Alginate–lavender nanofibers with antibacterial and anti-inflammatory activity to effectively promote burn healing. J. Mater. Chem. B.

[17] Monou P.K., Mamaligka A.M., Tzimtzimis E.K., Tzetzis D., Vergkizi-Nikolakaki S., Vizirianakis I.S., Andriotis E.G., Eleftheriadis G.K., Fatouros D.G. (2022). Fabrication and preliminary in vitro evaluation of 3D-printed alginate films with cannabidiol (CBD) and cannabigerol (CBG) nanoparticles for potential wound-healing applications. Pharmaceutics.

[18] Chelminiak-Dudkiewicz D., Machacek M., Dlugaszewska J., Wujak M., Smolarkiewicz-Wyczachowski A., Bocian S., Mylkie K., Goslinski T., Marszall M.P., Ziegler-Borowska M. (2023). Fabrication and characterization of new levan@CBD biocomposite sponges as potential materials in natural, non-toxic wound dressing applications. Int. J. Biol. Macromol..

[19] Pisanti S., Malfitano A.M., Ciaglia E., Lamberti A., Ranieri R., Cuomo G., Abate M., Faggiana G., Proto M.C., Fiore D., Laezza C., Bifulco M. (2017). Cannabidiol: state of the art and new challenges for therapeutic applications. Pharmacol. Ther..

[20] Río C.d., Millán E., García V., Appendino G., DeMesa J., Muñoz E. (2018). The endocannabinoid system of the skin. A potential approach for the treatment of skin disorders. Biochem. Pharmacol..

[21] Sheriff T., Lin M.J., Dubin D., Khorasani H. (2020). The potential role of cannabinoids in dermatology. J. Dermatol. Treat..

[22] Raj V., Park J.G., Cho K.-H., Choi P., Kim T., Ham J., Lee J. (2021). Assessment of antiviral potencies of cannabinoids against SARS-CoV-2 using computational and in vitro approaches. Int. J. Biol. Macromol..

[23] Chełminiak-Dudkiewicz D., Smolarkiewicz-Wyczachowski A., Ziegler-Borowska M., Kaczmarek H. (2024). Photochemical stability of chitosan films doped with cannabis oil. J. Photochem. Photobiol. B Biol..

[24] Choi S.Y., Yoon K.-h., Lee J.I., Mitchell R.J. (2015). Violacein: properties and production of a versatile bacterial pigment. BioMed Res. Int..

[25] Pauer H., Hardoim C.C.P., Teixeira F.L., Miranda K.R., Barbirato D.d.S., Carvalho D.P.d., Antunes L.C.M., Leitão Á.A.d.C., Lobo L.A., Domingues R.M.C.P. (2018). Impact of violacein from Chromobacterium violaceum on the mammalian gut microbiome. PLoS One.

[26] Park H., Park S., Yang Y.-H., Choi K.-Y. (2021). Microbial synthesis of violacein pigment and its potential applications. Crit. Rev. Biotechnol..

[27] Rivero Berti I., Gantner M.E., Rodriguez S., Islan G.A., Fávaro W.J., Talevi A., Castro G.R., Durán N. (2023). Potential biocide roles of violacein. Frontiers in Nanotechnology.

[28] Duran N., Justo G., Nakazato G., Fávaro W., Violacein (2022). A microbial antiviral product: does play key role as active agent against SARS-CoV-2?. Int. J. Med. Rev..

[29] Wegrzynowska-Drzymalska K., Grebicka P., Mlynarczyk D.T., Chelminiak-Dudkiewicz D., Kaczmarek H., Goslinski T., Ziegler-Borowska M. (2020). Crosslinking of chitosan with dialdehyde chitosan as a new approach for biomedical applications. Materials.

[30] Liu Y., Cai Z., Sheng L., Ma M., Xu Q., Jin Y. (2019). Structure-property of crosslinked chitosan/silica composite films modified by genipin and glutaraldehyde under alkaline conditions. Carbohydr. Polym..

[31] Fan Y., Lu Q., Liang W., Wang Y., Zhou Y., Lang M. (2021). Preparation and characterization of antibacterial polyvinyl alcohol/chitosan sponge and potential applied for wound dressing. Eur. Polym. J..

[32] Chen W., Li X., Zeng L., Pan H., Liu Z. (2021). Allicin-loaded chitosan/polyvinyl alcohol scaffolds as a potential wound dressing material to treat diabetic wounds: an in vitro and in vivo study. J. Drug Deliv. Sci. Technol..

[33] Tak U.N., Rashid S., Kour P., Nazir N., Zargar M.I., Dar A.A. (2023). Bergenia stracheyi extract-based hybrid hydrogels of biocompatible polymers with good adhesive, stretching, swelling, self-healing, antibacterial, and antioxidant properties. Int. J. Biol. Macromol..

[34] He Y., Zhao W., Dong Z., Ji Y., Li M., Hao Y., Zhang D., Yuan C., Deng J., Zhao P., Zhou Q. (2021). A biodegradable antibacterial alginate/carboxymethyl chitosan/Kangfuxin sponges for promoting blood coagulation and full-thickness wound healing. Int. J. Biol. Macromol..

[35] Bozin B., Mimica-Dukic N., Simin N., Anackov G. (2006). Characterization of the volatile composition of essential oils of some lamiaceae spices and the antimicrobial and antioxidant activities of the entire oils. J. Agric. Food Chem..

[36] Nowak A., Zagórska-Dziok M., Perużyńska M., Cybulska K., Kucharska E., Ossowicz-Rupniewska P., Piotrowska K., Duchnik W., Kucharski Ł., Sulikowski T., Droździk M., Klimowicz A. (2022). Assessment of the anti-inflammatory, antibacterial and anti-aging properties and possible use on the skin of hydrogels containing epilobium angustifolium L. Extracts. Front. Pharmacol..

[37] Chelminiak-Dudkiewicz D., Smolarkiewicz-Wyczachowski A., Mylkie K., Wujak M., Mlynarczyk D.T., Nowak P., Bocian S., Goslinski T., Ziegler-Borowska M. (2022). Chitosan-based films with cannabis oil as a base material for wound dressing application. Sci. Rep..

[38] Chełminiak-Dudkiewicz D., Ziegler-Borowska M., Stolarska M., Sobotta L., Falkowski M., Mielcarek J., Goslinski T., Kowalonek J., Węgrzynowska-Drzymalska K., Kaczmarek H. (2018). The chitosan – porphyrazine hybrid materials and their photochemical properties. J. Photochem. Photobiol. B Biol..

[39] Islam M.M., Islam R., Mahmudul Hassan S.M., Karim M.R., Rahman M.M., Rahman S., Nur Hossain M., Islam D., Aftab Ali Shaikh M., Georghiou P.E. (2023). Carboxymethyl chitin and chitosan derivatives: synthesis, characterization and antibacterial activity. Carbohydrate Polymer Technologies and Applications.

[40] Li H., Zhao Q.-S., Chang S.-L., Chang T.-R., Tan M.-H., Zhao B. (2022). Development of cannabidiol full-spectrum oil/2,6-di-O-methyl-β-cyclodextrin inclusion complex with enhanced water solubility, bioactivity, and thermal stability. J. Mol. Liq..

[41] Anahas A.M.P., Kumaran S., Kandeel M., Muralitharan G., Silviya J., Adhimoolam G.L., Panagal M., Pugazhvendan S.R., Suresh G., Wilson Aruni A., Rethinam S., Prasannabalaji N. (2022). Applications of natural violet pigments from halophilic *Chromobacterium violaceum* PDF23 for textile dyeing with antimicrobial and antioxidant potentials. J. Nanomater..

[42] Subramaniam S., Ravi V., Sivasubramanian A. (2014). Synergistic antimicrobial profiling of violacein with commercial antibiotics against pathogenic micro-organisms. Pharmaceut. Biol..

[43] Luo Y., Cui L., Zou L., Zhao Y., Chen L., Guan Y., Zhang Y. (2022). Mechanically strong and on-demand dissoluble chitosan hydrogels for wound dressing applications. Carbohydr. Polym..

[44] Li Q., Lu F., Zhou G., Yu K., Lu B., Xiao Y., Dai F., Wu D., Lan G. (2017). Silver inlaid with gold nanoparticle/chitosan wound dressing enhances antibacterial activity and porosity, and promotes wound healing. Biomacromolecules.

[45] Akartasse N., Azzaoui K., Mejdoubi E., Elansari L.L., Hammouti B., Siaj M., Jodeh S., Hanbali G., Hamed R., Rhazi L. (2022). Chitosan-Hydroxyapatite bio-based composite in film form: synthesis and application in wastewater. Polymers.

[46] Magli S., Rossi L., Consentino C., Bertini S., Nicotra F., Russo L. (2021). Combined analytical approaches to standardize and characterize biomaterials formulations: application to chitosan-gelatin cross-linked hydrogels. Biomolecules.

[47] Almaieli L.M.A., Khalaf M.M., Gouda M., Elmushyakhi A., Abou Taleb M.F., Abd El-Lateef H.M. (2023). Fabrication of bio-based film comprising metal oxide nanoparticles loaded chitosan for wound dressing applications. Polymers.

[48] Ho T.T.-P., Doan V.K., Tran N.M.-P., Nguyen L.K.-K., Le A.N.-M., Ho M.H., Trinh N.-T., Van Vo T., Tran L.D., Nguyen T.-H. (2021). Fabrication of chitosan oligomer-coated electrospun polycaprolactone membrane for wound dressing application. Mater. Sci. Eng. C.

[49] Jiang S., Deng J., Jin Y., Qian B., Lv W., Zhou Q., Mei E., Neisiany R.E., Liu Y., You Z., Pan J. (2023). Breathable, antifreezing, mechanically skin-like hydrogel textile wound dressings with dual antibacterial mechanisms. Bioact. Mater..

[50] Rafieian S., Mahdavi H., Masoumi M.E. (2019). Improved mechanical, physical and biological properties of chitosan films using Aloe vera and electrospun PVA nanofibers for wound dressing applications. J. Ind. Textil..

[51] Genevro G.M., Neto R.J.G., de Almeida Paulo L., Lopes P.S., de Moraes M.A., Beppu M.M. (2020). Erratum to: glucomannan asymmetric membranes for wound dressing–Erratum. J. Mater. Res..

[52] Samadi A., Azandeh S., Orazizadeh M., Bayati V., Rafienia M., Karami M.A. (2021). Fabrication and characterization of glycerol/chitosan/polyvinyl alcohol-based transparent hydrogel films loaded with silver nanoparticles for antibacterial wound dressing applications. Adv. Biomed. Res..

[53] Lin C.-M., Chang Y.-C., Cheng L.-C., Liu C.-H., Chang S.C., Hsien T.-Y., Wang D.-M., Hsieh H.-J. (2020). Preparation of graphene-embedded hydroxypropyl cellulose/chitosan/polyethylene oxide nanofiber membranes as wound dressings with enhanced antibacterial properties. Cellulose.

[54] Sulastri E., Zubair M.S., Lesmana R., Mohammed A.F.A., Wathoni N. (2021). Development and characterization of ulvan polysaccharides-based hydrogel films for potential wound dressing applications. Drug Des. Dev. Ther..

[55] Rezvani E., Schleining G., Sümen G., Taherian A.R. (2013). Assessment of physical and mechanical properties of sodium caseinate and stearic acid based film-forming emulsions and edible films. J. Food Eng..

[56] Wang D., Zhang N., Meng G., He J., Wu F. (2020). The effect of form of carboxymethyl-chitosan dressings on biological properties in wound healing. Colloids Surf. B Biointerfaces.

[57] Pansara C., Mishra R., Mehta T., Parikh A., Garg S. (2020). Formulation of chitosan stabilized silver nanoparticle-containing wound healing film: in vitro and in vivo characterization. J. Pharmaceut. Sci..

[58] Saberian M., Seyedjafari E., Zargar S.J., Mahdavi F.S., Sanaei-rad P. (2021). Fabrication and characterization of alginate/chitosan hydrogel combined with honey and aloe vera for wound dressing applications. J. Appl. Polym. Sci..

[59] Lin N., Zuo B. (2021). Silk sericin/fibroin electrospinning dressings: a method for preparing a dressing material with high moisture vapor transmission rate. J. Biomater. Sci. Polym. Ed..

[60] Minsart M., Van Vlierberghe S., Dubruel P., Mignon A. (2022). Commercial wound dressings for the treatment of exuding wounds: an in-depth physico-chemical comparative study. Burns & Trauma.

[61] do Nascimento M.F., Cardoso J.C., Santos T.S., Tavares L.A., Pashirova T.N., Severino P., Souto E.B., Albuquerque-Junior R.L.C. (2020). Development and characterization of biointeractive gelatin wound dressing based on extract of punica granatum linn. Pharmaceutics.

[62] Tamahkar E., Özkahraman B., Özbaş Z., İzbudak B., Yarimcan F., Boran F., Öztürk A.B. (2021). Aloe vera-based antibacterial porous sponges for wound dressing applications. J. Porous Mater..

[63] Biranje S.S., Madiwale P.V., Patankar K.C., Chhabra R., Dandekar-Jain P., Adivarekar R.V. (2019). Hemostasis and anti-necrotic activity of wound-healing dressing containing chitosan nanoparticles. Int. J. Biol. Macromol..

[64] Hashemi Doulabi A., Mirzadeh H., Imani M., Bagheri-Khoulenjani S. (2018). Chitosan/polyethylene glycol fumarate blend films for wound dressing application: in vitro biocompatibility and biodegradability assays. Progress in Biomaterials.

[65] Lungu R., Paun M.-A., Peptanariu D., Ailincai D., Marin L., Nichita M.-V., Paun V.-A., Paun V.-P. (2022). Biocompatible chitosan-based hydrogels for bioabsorbable wound dressings. Gels.

[66] Ratajska M., Strobin G., Wiśniewska-Wrona M., Ciechańska D., Struszczyk H., Boryniec S., Biniaś D., Biniaś W. (2003). Studies on the biodegradation of chitosan in an aqueous medium. Fibres Text. East. Eur..

[67] Wei Q., Chen K., Zhang X., Ma G., Zhang W., Hu Z. (2022). Facile preparation of polysaccharides-based adhesive hydrogel with antibacterial and antioxidant properties for promoting wound healing. Colloids Surf. B Biointerfaces.

[68] Xiong S., Li R., Ye S., Ni P., Shan J., Yuan T., Liang J., Fan Y., Zhang X. (2022). Vanillin enhances the antibacterial and antioxidant properties of polyvinyl alcohol-chitosan hydrogel dressings. Int. J. Biol. Macromol..

[69] Nafis A., Kasrati A., Jamali C.A., Mezrioui N., Setzer W., Abbad A., Hassani L. (2019). Antioxidant activity and evidence for synergism of Cannabis sativa (L.) essential oil with antimicrobial standards. Ind. Crop. Prod..

[70] Cheng K.-C., Hsiao H.-C., Hou Y.-C., Hsieh C.-W., Hsu H.-Y., Chen H.-Y., Lin S.-P. (2022). Improvement in violacein production by utilizing formic acid to induce quorum sensing in Chromobacterium violaceum. Antioxidants.

[71] Huang C., Chu X., Hui W., Xie C., Xu X. (2023). Study on extraction and characterization of new antibiotics violacein from engineered Escherichia coli VioABCDE-SD. Biotechnol. Appl. Biochem..

[72] Bölgen N., Demir D., Yalçın M.S., Özdemir S. (2020). Development of Hypericum perforatum oil incorporated antimicrobial and antioxidant chitosan cryogel as a wound dressing material. Int. J. Biol. Macromol..

[73] Tamer T.M., ElTantawy M.M., Brussevich A., Nebalueva A., Novikov A., Moskalenko I.V., Abu-Serie M.M., Hassan M.A., Ulasevich S., Skorb E.V. (2023). Functionalization of chitosan with poly aromatic hydroxyl molecules for improving its antibacterial and antioxidant properties: practical and theoretical studies. Int. J. Biol. Macromol..

[74] Dharmadeva S., Galgamuwa L.S., Prasadinie C., Kumarasinghe N. (2018). In vitro anti-inflammatory activity of Ficus racemosa L. bark using albumin denaturation method. Ayu.

[75] Argel S., Castaño M., Jimenez D.E., Rodríguez S., Vallejo M.J., Castro C.I., Osorio M.A. (2022). Assessment of bacterial nanocellulose loaded with acetylsalicylic acid or povidone-iodine as bioactive dressings for skin and soft tissue infections. Pharmaceutics.

[76] Ali A., Bano S., Poojary S.S., Priyadarshi R., Choudhary A., Kumar D., Negi Y.S. (2022). Comparative analysis of TiO2 and Ag nanoparticles on xylan/chitosan conjugate matrix for wound healing application. International Journal of Polymeric Materials and Polymeric Biomaterials.

[77] Shitole A.A., Raut P., Giram P., Rade P., Khandwekar A., Garnaik B., Sharma N. (2020). Poly (vinylpyrrolidone)-iodine engineered poly (ε-caprolactone) nanofibers as potential wound dressing materials. Mater. Sci. Eng. C.

[78] Kaur T., Thirugnanam A. (2016). Tailoring in vitro biological and mechanical properties of polyvinyl alcohol reinforced with threshold carbon nanotube concentration for improved cellular response. RSC Adv..

[79] Shitole A.A., Raut P.W., Sharma N., Giram P., Khandwekar A.P., Garnaik B. (2019). Electrospun polycaprolactone/hydroxyapatite/ZnO nanofibers as potential biomaterials for bone tissue regeneration. J. Mater. Sci. Mater. Med..

[80] WHO, Global Guidelines for the Prevention of Surgical Site Infection (2018).

[81] Roy S., Santra S., Das A., Dixith S., Sinha M., Ghatak S., Ghosh N., Banerjee P., Khanna S., Mathew-Steiner S., Ghatak P.D., Blackstone B.N., Powell H.M., Bergdall V.K., Wozniak D.J., Sen C.K. (2020). Staphylococcus aureus biofilm infection compromises wound healing by causing deficiencies in granulation tissue collagen. Ann. Surg..

[82] Ruhaak L.R., Smit N.P.M., Romijn F.P.H.T.M., Pieterse M.M., van der Laarse A., van der Burgt Y.E.M., Cobbaert C.M. (2018). Robust and accurate 2-year performance of a quantitative mass spectrometry-based apolipoprotein test in a clinical chemistry laboratory. Clin. Chem..

[83] Raj V., Kim Y., Kim Y.-G., Lee J.-H., Lee J. (2022). Chitosan-gum Arabic embedded alizarin nanocarriers inhibit biofilm formation of multispecies microorganisms. Carbohydr. Polym..

[84] Johnson B. (2005).

[85] Bakun P., Czarczynska-Goslinska B., Mlynarczyk D.T., Musielak M., Mylkie K., Dlugaszewska J., Koczorowski T., Suchorska W.M., Ziegler-Borowska M., Goslinski T., Krakowiak R. (2022). Gallic acid-functionalized, TiO2-based nanomaterial—preparation, physicochemical and biological properties. Materials.

[86] Yilmaz Atay H., Jana S., Jana S. (2019). Functional Chitosan: Drug Delivery and Biomedical Applications.

[87] Schofs L., Sparo M.D., Sánchez Bruni S.F. (2021). The antimicrobial effect behind Cannabis sativa. Pharmacol Res Perspect.

[88] Blaskovich M.A.T., Kavanagh A.M., Elliott A.G., Zhang B., Ramu S., Amado M., Lowe G.J., Hinton A.O., Pham D.M.T., Zuegg J., Beare N., Quach D., Sharp M.D., Pogliano J., Rogers A.P., Lyras D., Tan L., West N.P., Crawford D.W., Peterson M.L., Callahan M., Thurn M. (2021). The antimicrobial potential of cannabidiol. Commun. Biol..

[89] Silva M.D., Paris J.L., Gama F.M., Silva B.F.B., Sillankorva S. (2021). Sustained release of a Streptococcus pneumoniae endolysin from liposomes for potential otitis media treatment. ACS Infect. Dis..

[90] Gildea L., Ayariga J.A., Xu J., Villafane R., Robertson B.K., Samuel-Foo M., Ajayi O.S. (2022). Cannabis sativa CBD extract exhibits Synergy with broad-spectrum antibiotics against Salmonella enterica subsp. enterica serovar typhimurium. Microorganisms.

[91] Backhaus T., Grimme L.H. (1999). The toxicity of antibiotic agents to the luminescent bacterium Vibrio fischeri. Chemosphere.

[92] Maliński M.P., Budzianowski J., Kikowska M., Derda M., Jaworska M.M., Mlynarczyk D.T., Szukalska M., Florek E., Thiem B. (2021). Two ecdysteroids isolated from micropropagated lychnis flos-cuculi and the biological activity of plant material. Molecules.

