# Supplementary material for: Enhancing the porosity of chitosan sponges with CBD by adding antimicrobial violacein
Source: Heliyon. 2024 Jul 31;10(15):e35389. doi: 10.1016/j.heliyon.2024.e35389 (PMC11334822; doi:10.1016/j.heliyon.2024.e35389)
Supplement: Multimedia component 1 [file mmc1.docx]

Supplementary Material

Enhancing the porosity of chitosan sponges with CBD by adding antimicrobial violacein

Dorota Chełminiak-Dudkiewicz^1*^, Magdalena Wujak^2^, Dariusz Młynarczyk^3^, Jolanta Długaszewska^4^, Kinga Mylkie^1^, Marta Ziegler-Borowska^1^

^1^ Department of Biomedical Chemistry and Polymer Science, Faculty of Chemistry, Nicolaus Copernicus University in Torun, Gagarina 7, 87-100 Torun, Poland

^2^ Department of Medicinal Chemistry, Faculty of Pharmacy, Collegium Medicum in Bydgoszcz, Nicolaus Copernicus University in Torun, Jurasza 2, 85-089 Bydgoszcz, Poland

^3^ Chair and Department of Chemical Technology of Drugs, Poznan University of Medical Sciences, Grunwaldzka 10, 60-780 Poznan, Poland

^4^ Department of Genetics and Pharmaceutical Microbiology, Poznan University of Medical Sciences, Rokietnicka 3, 60-806 Poznan, Poland

**Table S1.** Thermal parameters of the obtained materials.

| **Sample** | **First stage** | | **Second stage** | | | **Third stage** | | | **Fourth stage** | | | **Residue at 600°C (%)** |
| --- | --- | --- | --- | --- | --- | --- | --- | --- | --- | --- | --- | --- |
|  | *T*_max_(°C) | Δ*m* (*%*) | *T*_0_ (°C) | *T*_max_ (°C) | Δ*m* (*%*) | *T*_0_ (°C) | *T*_max_(°C) | Δ*m* (*%*) | *T*_0_ (°C) | *T*_max_(°C) | Δ*m* (*%*) |  |
| **CS** | 58 | 9 | 90 | 139 | 14 | 216 | 275 | 52 | - | - | - | 25 |
| **(CanO-Viol)CS** | 61 | 7 | 96 | 136 | 13 | 213 | 285 | 34 | 342 | 398 | 28 | 18 |
